# Air Pollution in a Northwest Chinese Valley City (2020–2024): Integrated WRF-HYSPLIT Modeling of Pollution Characteristics, Meteorological Drivers, and Transport Pathways in Yining

**DOI:** 10.3390/toxics13100868

**Published:** 2025-10-13

**Authors:** Xiaoqi Liu, Wei Wen, Xin Ma, Dayi Qian, Weiqing Zhang, Shaorui Wang

**Affiliations:** 1 School of Energy and Environmental Engineering, University of Science and Technology Beijing, Beijing 100083, China; 18513216926@163.com (X.L.); qday@ustb.edu.cn (D.Q.); m202520245@xs.ustb.edu.cn (S.W.); 2 CMA Earth System Modeling and Prediction Centre (CEMC), Beijing 100081, China; 3 Xinjiang Key Laboratory of Clean Conversion and High Value Utilization of Biomass Resources, Yili Normal University, Yining 835000, China; 4 Yining Environmental Monitoring Station, Yining 835000, China; 15909997016@163.com

**Keywords:** air pollution, valley city, meteorological drivers, transport pathways

## Abstract

This study investigates the characteristics, meteorological drivers, and transport pathways of air pollution in Yining City from 2020 to 2024 based on meteorological records and air pollutant monitoring data. An integrated modeling approach combining the Weather Research and Forecasting (WRF) model and the Hybrid Single-Particle Lagrangian Integrated Trajectory (HYSPLIT) model was employed. Results reveal an overall annual decrease in ambient pollutant concentrations in Yining, with PM_2.5_ and PM_10_ consistently below the national secondary standards, In contrast, the O_3_ concentration shows a marked yearly increase. Pronounced seasonal variations were identified: the elevated O_3_ concentrations in summer were driven by high temperatures and intense solar radiation. The significant increase in PM_2.5_ and PM_10_ concentrations during winter was predominantly attributed to coal-based heating emissions and temperature inversion conditions. Pollutant concentrations were strongly associated with gaseous precursors (e.g., CO and NO_2_) and meteorological factors. Higher temperatures and lower relative humidity aggravated O_3_ formation, whereas lower temperatures and higher relative humidity favored PM_2.5_ pollution. Correlation analysis revealed that NO_2_ and CO showed the strongest correlations with PM_2.5_ (r = 0.84) and O_3_ (r = −0.62), respectively. Backward trajectory analysis revealed that higher pollution levels were associated with air masses originating from the southwest and southeast.

## 1. Introduction

With rapid industrialization and urbanization, cities have experienced a substantial increase in energy consumption and air pollutant emissions, leading to increasingly serious environmental challenges. Consequently, air pollution prevention and control have become a crucial aspect of China’s ecological civilization initiatives. Air quality exerts a profound influence on public health; for instance, PM_2.5_ can penetrate deeply into the respiratory tract and enter the bloodstream, elevating the risks of cardiovascular diseases, diabetes, lung cancer, and stroke [1]. Similarly, exposure to ozone is associated with respiratory symptoms such as chest tightness, shortness of breath, and throat irritation, while chronic exposure may have also contributed to ocular damage and visual impairment [2].

In recent years, extensive research has been conducted on the drivers of air pollution and corresponding mitigation strategies across China. However, the majority of these studies have concentrated on economically developed areas, such as the Beijing–Tianjin–Hebei region [3], the Yangtze River Delta [4], and the Pearl River Delta [5]. As a result of industrial relocation policies, many pollution-intensive industries have shifted to western China, such as the Xinjiang Uygur Autonomous Region, where environmental standards are more relaxed [6]. This transition has spurred rapid urban population growth and industrial agglomerations, significantly raising local emissions [7]. Although these developments have stimulated employment and economic growth, they have also exacerbated air pollution.

Yining City, the administrative capital of the Ili Kazakh Autonomous Prefecture, is situated in the Xinjiang Uygur Autonomous Region of northwestern China. It lies within Ili River Valley, a north–south trending basin flanked by the Tianshan Mountains to the north and south, forming a semi-enclosed topography setting [8]. With an average elevation of 1080 m, Yining’s urban area occupies the transitional zone between the mountain foothills and the river valley plain. This geographical layout significantly restricts the atmospheric diffusion, particularly during the heating season [9]. Yining City has a moderate continental climate, with an annual precipitation of 245.1 mm and a mean temperature of 10.5 °C, marked by considerable diurnal temperature variation. Temperature inversions are frequently (83% probability) during the heating season, with an average thickness of 511 m. Ground-level and multi-layer inversions dominate [10]. These conditions—combined with generally low wind speeds—aggravate the poor natural dispersal of air pollutants. Furthermore, recent industrial development in Yining has emphasized energy-intensive sectors, including coal-fired power generation, coal chemical processing, biopharmaceuticals, and advanced construction materials [11], resulting in significant deterioration of air quality [12]. Monitoring data from 2015 to 2020 show variable pollution levels in Yining City: after an initial rise, concentrations of PM_10_ and PM_2.5_ began to decline, yet remained consistently above China’s Grade II air quality standards. By 2020, PM_10_ and PM_2.5_ concentrations reached 71 μg/m^3^ and 43 μg/m^3^, respectively, up by 4.4% and 16.2% from 2015, respectively. In the same period, O_3_ concentrations rose consistently from 70 μg/m^3^ to 83 μg/m^3^, an increase of 18.6%. Although SO_2_ and CO levels showed a downward trend, Yining City still recorded the highest regional concentrations in Xinjiang, with SO_2_ at 14 μg/m^3^ and CO concentrations persistently elevated in recent years [13]. Pollution exceedance days in Yining City are classified as light, moderate, or severe, with moderate to severe episodes displaying clear seasonality concentrated mainly in the heating season. The city’s unique topography results in distinctly regional pollution patterns. Unlike flat terrains, the valley’s restricted airflow greatly diminishes the atmosphere’s pollutant carrying capacity, especially in winter, intensifying dispersion challenges. Therefore, identifying the drivers of air pollution in Yining City, analyzing valley-specific pollution characteristics, clarifying transport pathways, and pinpointing potential source regions are of considerable importance for formulating effective control strategies.

Research on air pollution has largely focused on economically developed regions characterized by intensive industrial activity. For instance, Zhang et al. [14] systematically investigated the regional heterogeneity of summertime O_3_ pollution in Beijing in 2018 by integrating the Community Multiscale Air Quality modeling system (CMAQ) with process analysis. Their work quantified source contributions and formation mechanisms, revealing that non-local emissions from surrounding regions had a significantly greater impact on O_3_ concentrations than urban and suburban sources. Similarly, Ge et al. [15] employed the Comprehensive Air Quality Model with Extensions with the Ozone Source Apportionment Technology (CAMx-OSAT) to simulate ozone reduction scenarios in Texas, concluding that controlling high-altitude point source emissions was more effective for ozone abatement than reducing road emissions. Liang et al. [16] employed the WRF-CAMx with Particulate Source Apportionment Technology (PSAT) to assess the impact of vehicle emission on air quality in the Beijing–Tianjin–Hebei region, demonstrating the significant role of non-local pollution transport. In a related study, Wen et al. [17] utilized the CAMx model to conduct the source apportionment of atmospheric pollutants in Beijing. Regarding the complex topography characteristic of northwestern China, Yang et al. [18] investigated air pollution patterns in the valley city of Lanzhou. They identified particulate matter as the dominant pollutant in Xigu District, with emissions showing strong seasonality—highest in winter due to increased emissions and poor dispersion, and lower in summer. O_3_ levels varied seasonally, with higher levels observed in spring compared to summer. This pattern was attributed to higher NOx precursor concentrations and weaker atmospheric diffusion during spring. However, research on air pollution sources in Xinjiang remains limited. The transport mechanisms of pollutants in the Tianshan region is considerably more complex than in plain areas due to mountainous terrain and more dynamic meteorological conditions [19].

As a typical valley city, Yining’s unique topography and meteorology leads to the accumulation of air pollutants. However, systematic studies on its pollution sources and key drivers are still lacking. With the city undergoing rapid development, improving regional air quality has become an urgent priority. This study investigates air pollution in Yining through an integrated analysis of meteorological and air quality monitoring data, combined with a customized atmospheric numerical model for the region. It systematically explores the characteristics, formation mechanisms, and influencing factors of air pollution in valley cities, aiming to provide scientific support for Yining’s pollution control strategies. The results are also expected to offer valuable references for air quality management in other topographically similar regions.

## 2. Materials and Methods

### 2.1. Study Area Description

**Figure 1:**
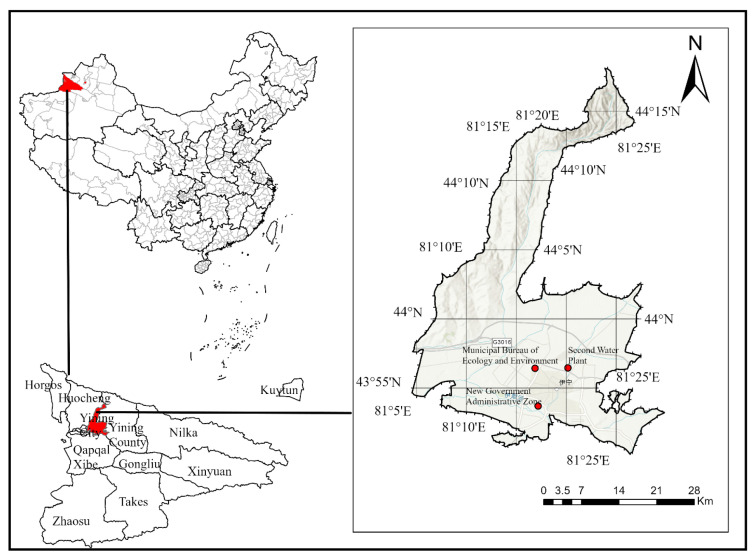
Map showing the geographical location of the study area. The three red dots indicate the air quality monitoring stations in Yining City.

Yining City, the administrative center of Ili Kazakh Autonomous Prefecture, is situated in the northwestern part of Xinjiang Uygur Autonomous Region, China (43°50′–44°09′ N, 81°04′–81°29′ E). As the western terminus of China National Highway 312, the city serves as a crucial transportation junction. The city is bordered by Yining County to the east, Huocheng County to the west, Qapqal Xibe Autonomous County to the south-separated by a river boundary, and is backed by the Kogurqin Mountains, a branch range of the Tianshan Mountains, to the north. It lies within the Ili River Valley of the northern Tianshan Mountains, characterized by a distinct valley topography with low-lying terrain enclosed by mountains on three sides (as shown in Figure 1). The city covers an administrative area of 524.94 km^2^. In 2024, the city’s economy grew by 6.1% year-on-year, with its gross domestic product (GDP) reaching CNY 42.691 billion. Sectoral analysis reveals a tertiary-sector-dominated industrial structure: the tertiary sector contributed CNY 27.314 billion (64.0%), followed by the secondary sector at CNY 14.248 billion (33.4%), and the primary sector at CNY 1.129 billion (2.6%). Pollutant source census data from 2020 show that emissions of SO_2_ and NO_x_ in Yining are 18,533.43 t and 42,480.28 t, which were dominated by stationary combustion sources (accounting for 82.0% and 71.5%, respectively), industrial process sources (16.1% and 5.9%), and mobile sources (1.1% and 21.1%). The emission of atmospheric particulate matter is 7197.84 t, which was dominated by stationary combustion sources (62.9%), cooking fume sources (11.9%), and fugitive dust sources (9.9%). The emission of volatile organic compounds (VOCs) is 11,657.36 t, which was mainly dominated by stationary combustion sources (48.2%), mobile sources (15.8%), and natural sources (12.7%).

### 2.2. Data Source

This research analyzed the daily average air pollutant concentrations (PM_2.5_, PM_10_, SO_2_, NO_2_, CO, and O_3_) in Yining City. Pollution data were obtained from the Environmental Meteorological Service Platform (http://eia-data.com (accessed on 1 May 2025)), which aggregates measurements from three national-level automatic air quality monitoring stations: the Municipal Environmental Protection Bureau station (43.94° N, 81.28° E), the Second Water Treatment Plant station (43.94° N, 81.34° E), and the New Administrative District station (43.89° N, 81.29° E). The spatial distribution of these stations is shown in Figure 1. All stations operate in strict accordance with China’s Ambient Air Quality Standards (GB 3095-2012) [20]. This study collected daily average concentrations of PM_2.5_, PM_10_, SO_2_, NO_2_, CO, and O_3_ from 2020 to 2024, yielding 1825 initial samples. Following quality control screening to remove records with missing values, 1461 complete daily records were retained for analysis.

Meteorological data were primarily sourced from the China Meteorological Administration (CMA) “TianQing” Big Data Cloud Platform, with supplementary observations from the Yining City Meteorological Monitoring Station (43.9406° N, 81.3264° E). The meteorological dataset includes 2 m temperature, relative humidity, precipitation, and 10 m wind vector components (direction and speed). The number of valid meteorological records aligns with the air quality dataset, ensuring temporal consistency for subsequent correlation analysis.

### 2.3. Model Setup

**Figure 2:**
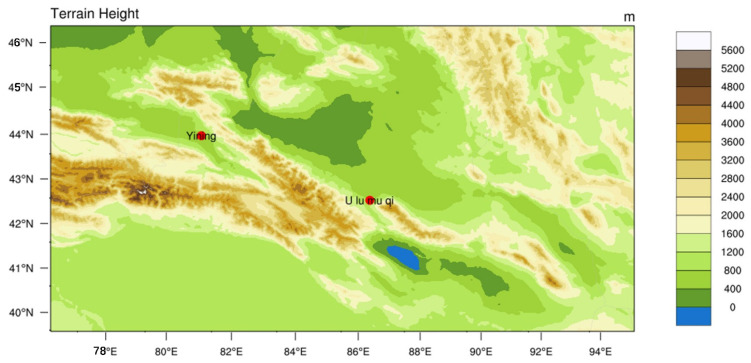
Modeling domain with terrain height profile.

To analyze pollution sources and transport pathways, this study established a coupled modeling system integrating the Weather Research and Forecasting (WRF v4.1) model and HYSPLIT-WEB trajectory model (NOAA HYSPLIT Model, https://www.ready.noaa.gov/hypub-bin/trajtype.pl?runtype=archive, (accessed on 1 May 2025)). The WRF simulation was configured with a Lambert conformal conic projection and a single-domain configuration centered at 44.1° N, 86.45° E. The modeling domain covered a 525 × 275 grid at 3 km horizontal resolution, encompassing Yining City and adjacent regions (Figure 2). Meteorological field simulation settings are summarized in Table 1. The WRF model was initialized using the Final Operational Global Analysis (FNL) dataset from the National Centers for Environmental Prediction (NCEP; http://rda.ucar.edu/datasets/ds083.2/, (accessed on 4 May 2025)) as boundary conditions. For pollution trajectory analysis, the HYSPLIT model was driven by NCEP reanalysis data. Given the potential for long-range transboundary transport affecting Yining City, the study selected the NCEP reanalysis dataset for its global coverage, long-term continuity, and direct compatibility with the HYSPLIT model.

This study employed the HYSPLIT backward trajectory model, an atmospheric dispersion simulation system co-developed by NOAA’s Air Resources Laboratory and the Australian Bureau of Meteorology [21]. Based on a Lagrangian framework for simulating advection and dispersion, the system incorporates multi-scale physical processes and heterogeneous emission sources within multi-parameter meteorological fields, enabling a comprehensive simulation of pollutant transport and transformation. As a well-validated numerical tool, HYSPLIT has been widely applied in regional to global studies of atmospheric transport patterns and transboundary pollution assessments [22,23,24].

### 2.4. Data Analysis Methodology

This study utilized Spearman correlation analysis to assess bivariate relationships between air pollutants and meteorological parameters. Based on a complete dataset of 1461 valid daily records, this study employed Spearman’s rank correlation analysis (*p* < 0.01) using SPSS Statistics 27.0.1 to quantify the relationships between atmospheric contaminants and meteorological factors. This analytical approach established empirical evidence to support pollution source attribution. As a symmetric statistical measure, correlation analysis identifies systemic relationships between variables without implying causation, distinguishing it from directional causal inference methods [25].

Additionally, this study integrated hourly meteorological observations (wind direction/speed) and concurrent pollution concentration data from Yining City (2020–2024) using polar coordinate overlay analysis. By mapping pollutant spatial distributions through color-gradient visualization superimposed on wind direction sector, the method enabled the quantitative evaluation of dispersion patterns as a function of wind speed and directions. This approach provided an effective technical basis for identifying regional pollution transport pathways by combining wind fields and contaminant distributions visualization.

## 3. Results and Discussion

### 3.1. Characteristics of Air Pollution in Yining City

**Figure 3:**
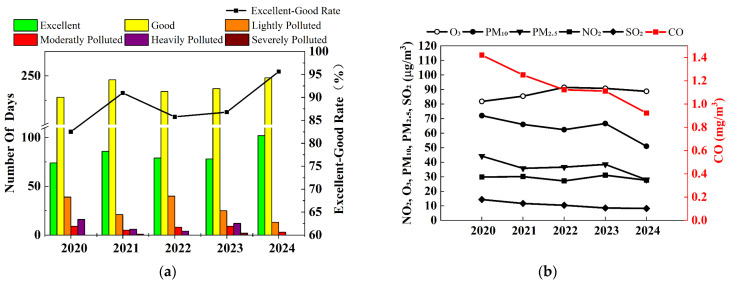
(**a**) Variation in air quality grade days in Yining City (2020–2024); (**b**) Interannual variations in six criteria pollutant concentrations in Yining City.

This study assessed the air quality status in Yining City from 2020 to 2024 using the Air Quality Index (AQI) methodology outlined in China’s HJ633-2012 technical standard [26]. The AQI is a dimensionless indicator used to quantitatively describe ambient air quality conditions, which is determined solely by the highest sub-index among the constituent pollutants. The AQI classification system defines six air quality levels: Excellent (0–50), Good (51–100), Lightly Polluted (101–150), Moderately Polluted (151–200), Heavily Polluted (201–300), and Severely Polluted (>300). The analysis revealed significant annual variations in air quality category distribution, as illustrated in Figure 3a.

During the study period, Yining City exhibited a fluctuating yet overall improving air quality. The annual proportion of days with excellent-to-good air quality (AQI ≤ 100) was 82.51% (302 days) in 2020, 90.96% (332 days) in 2021, 85.75% (313 days) in 2022, 86.30% (315 days) in 2023, and 95.63% (350 days) in 2024. The peak observed in 2020–2021 is likely attributable to COVID-19 control measures, such as industrial suspensions and transportation restrictions, while the subsequent rebound in 2022 coincided with the resumption of socioeconomic activities. Data from 2023 to 2024 indicate that air quality levels have been sustained beyond the levels observed during the pandemic period.

Overall, the excellent-to-good rate showed a gradual upward trend from 2020 to 2024, reflecting measurable enhancements in Yining City’s ambient air quality following the continuous implementation of national and local air pollution prevention and control measures.

Interannual variations in pollutant concentration were analyzed using 2020–2024 monitoring data collected from three air quality stations in Yining City (located at the Municipal Environmental Protection Bureau, Second Water Treatment Plant, and New Administrative District). As summarized in Table 2 and Figure 3b, SO_2_ and CO concentrations showed marked decreasing trends, while PM_2.5_ and PM_10_ exhibited fluctuating but overall declining patterns. NO_2_ levels remained relatively stable. These results reflect measurable improvements Yining’s ambient air quality during the five-year study period.

The annual average PM_2.5_ concentration demonstrated an overall decreasing trend, peaking at 44 μg/m^3^ in 2020 before declining to 36 μg/m^3^ in 2021. Subsequent rebounds elevated concentrations to 39 μg/m^3^ in 2023, followed by a further reduction to 28 μg/m^3^ in 2024, resulting in a cumulative decrease of 16 μg/m^3^ over the five-year period. Nevertheless, these levels consistently exceeded the 35 μg/m^3^ limit (except 2024) set in China’s Ambient Air Quality Standards (GB 3095-2012) [20], hereafter referred to as the Standards).

The annual average PM_10_ concentration followed a pattern similar to that of PM_2.5_, reaching a peak of 72 μg/m^3^ in 2020 and declining to a low of 51 μg/m^3^ in 2024, representing an overall decrease of 21 μg/m^3^. Concentrations remained above the 40 μg/m^3^ standard limit throughout the period. These results indicate that Yining City continues to face considerable particulate matter pollution, requiring further emission reductions to meet the primary annual air quality standard.

In contrast, SO_2_ pollution levels remain relatively low, with the five-year maximum concentration reaching only 14 μg/m^3^ in 2020—well below the 60 μg/m^3^ standard limit. SO_2_ concentrations declined consistently, reaching a minimum of 8 μg/m^3^ in 2024, a 43% reduction from 2020. Meanwhile, NO_2_ concentrations remained stable and below the 40 μg/m^3^ Standard threshold over the five-year period.

The annual average O_3_ concentration exhibited a trend opposite to that of particulate matter, showing an overall increase during the study period. Levels rose from 82 μg/m^3^ in 2020 to 91 μg/m^3^ in 2022, then declined gradually to 89 μg/m^3^ in 2024. Although still below the Standard’s 160 μg/m^3^ limit, the 2024 concentration was 7 μg/m^3^ higher than the 2020 baseline. Concurrently, CO concentrations decreased steadily, with the maximum recorded level of 1.4 mg/m^3^ in 2020 already below the 4 mg/m^3^ regulatory limit. By 2024, CO concentrations reached a minimum of 0.9 mg/m^3^, a reduction of 0.5 mg/m^3^ from 2020 levels.

The analysis of 2020–2024 monitoring data from Yining City’s three national air quality monitoring stations, evaluated against the Standards, reveals the percentage of exceedance days for each pollutant (Table 3). PM_2.5_ was the predominant pollutant, with an average of 12.039% of days exceeding the Standard—significantly higher than other pollutants. PM_10_ ranked second, with 5.144% of days exceeding the Standard over the five-year period. CO and NO_2_ showed lower exceedance rates of 3.502% and 1.916%, respectively, and no exceedances were recorded for either pollutant in 2024. This notable improvement confirms the effective implementation of Yining City’s Air Quality Attainment Plan (2021–2025), particularly through comprehensive industrial emission controls and multi-sector ultra-low emission initiatives. O_3_ showed an emerging trend of exceedance days during the five-year period, transitioning from zero occurrences to measurable levels, though overall frequency remained relatively low. Its formation is influenced by both meteorological conditions (particularly temperature and solar radiation) and gaseous precursor concentrations (notably VOCs, CO, and NOₓ). Therefore, future air pollution mitigation strategies should emphasize emission controls from stationary combustion and industrial process sources.

**Figure 4:**
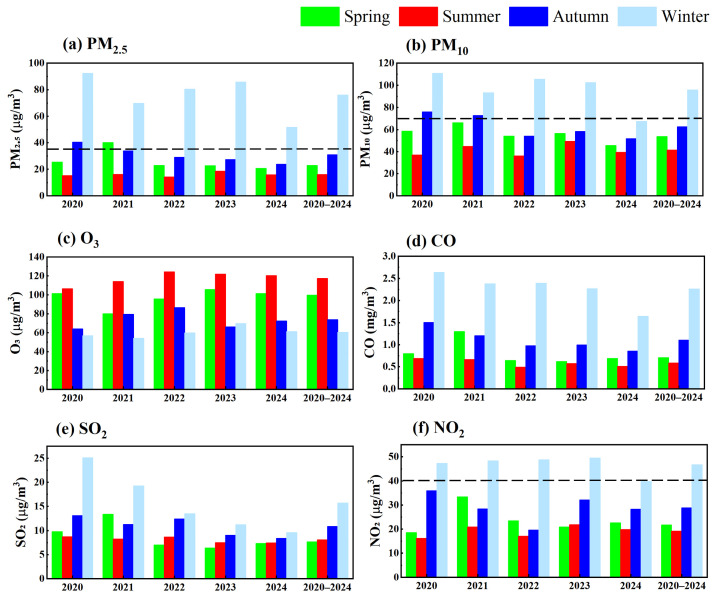
Seasonal variations in six criteria pollutants (**a**–**f**) in Yining City (2020–2024). The black dashed line denotes the secondary concentration standard, which is omitted for O_3_, CO and SO_2_ as their standard values exceed the upper bound of the axis scale.

PM_2.5_ concentrations exhibit distinct seasonal variability. Following the Northern Hemisphere climate patterns, the annual cycle is divided into winter (December–February), spring (March–May), summer (June–August), and autumn (September–November). Monthly averages from 2020 to 2024 reveal a consistent seasonal pattern (Figure 4a), with concentrations ranked as follows: winter (76 μg/m^3^) > autumn (31 μg/m^3^) > spring (23 μg/m^3^) > summer (16 μg/m^3^). Winter concentrations exceed other seasons by factors of 2.5–4.7, reflecting the highest pollution levels across the year. Similarly, PM_10_ follows a comparable seasons patterns (Figure 4b), with elevated concentrations in autumn and winter, ordered as winter (96 μg/m^3^) > autumn (62 μg/m^3^) > spring (54 μg/m^3^) > summer (41 μg/m^3^).

**Figure 5:**
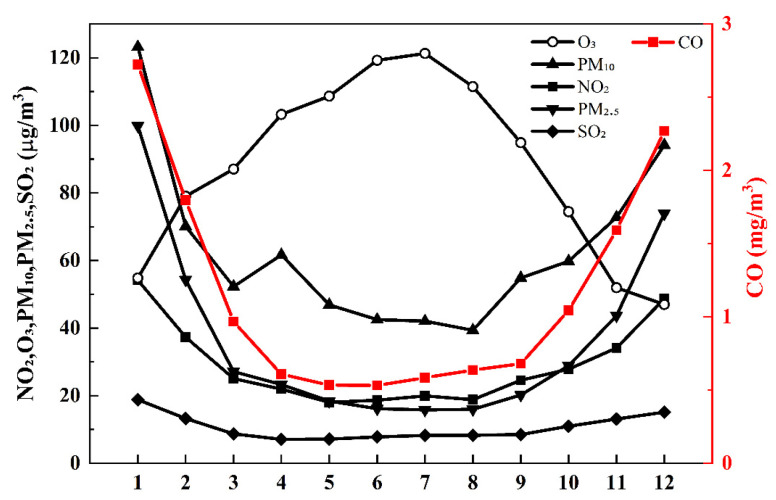
Monthly variations in six criteria pollutants in Yining City (2020–2024).

The analysis of monthly concentration data from 2020 to 2024 (Figure 5) revealed a characteristic U-shaped distribution, with the highest value occurring in January and December. This pattern reflects the combined influence of three key factors: winter heating demand, increased coal combustion emissions, and frequent temperature inversion events.

During the study period, PM_2.5_ concentrations reached an annual maximum in winter (mean 76 μg/m^3^) and gradually declined to their lowest levels in July–August (mean 16 μg/m^3^). Precipitation and other meteorological factors were identified as the primary drivers of this seasonal variation. The data showed consistently high concentrations in December each year. PM_10_ exhibited similar seasonal trends, with two notable exceptions: an anomalous concentration increase in April and an accelerated rebound in September. These anomalies are likely associated with transient particulate pollution events resulting from a transported dust storm. The monthly distributions of SO_2_, NO_2,_ and CO showed strong correlations with particulate matter trends. This correlation is attributable to increased emissions from coal-fired heating during winter months. In contrast, O_3_ displayed a distinct inverted U-shaped pattern, peaking at 119–121 μg/m^3^ in June–July. These summer maxima represent typical ozone formation conditions under elevated temperatures and strong solar radiation.

### 3.2. Effects of Meteorological Factors on Pollutants and Inter-Pollutant Correlations

**Figure 6:**
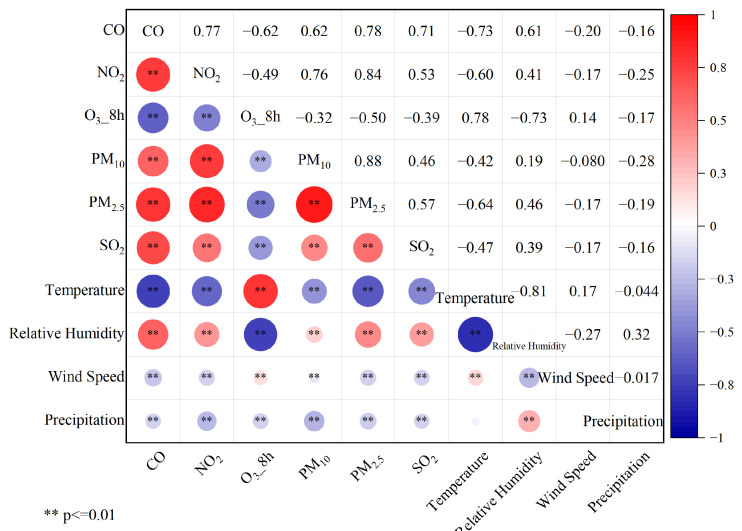
Pollutant–meteorology and pollutant–pollutant correlations.

The monthly variation patterns of pollutant concentrations in Yining City reveal that meteorological factors are a primary determinant of concentration fluctuations. Seasonal changes in meteorological conditions significantly influence air pollution levels, driving characteristic temporal patterns across different pollutants [27,28]. Key meteorological parameters, temperature and relative humidity mediate pollutant chemical transformation processes [29], while wind speed governs dispersion and transport dynamics [30]. Accordingly, this study performed correlation analyses between six pollutant categories and four meteorological variables (temperature, relative humidity, wind speed, and precipitation) utilizing 1461 valid samples. The results are summarized in Figure 6.

Regarding pollutant–meteorological relationships, CO concentrations exhibited significant negative correlations with temperature (r = −0.74), wind speed, and precipitation, but a strong positive correlation with relative humidity (r = 0.61). These patterns are likely attributable to the enhanced chemical conversion of CO at higher temperatures, resulting in lower concentrations.

NO_2_ concentrations showed correlation patterns similar to CO, with a significant negative correlation with temperature (r = −0.60) and a distinct positive correlation with relative humidity (r = 0.41). These relationships suggest that both temperature and relative humidity substantially influence NO_2_ concentrations, which consistently decrease with rising temperatures or declining humidity levels.

Similarly, SO_2_ concentrations showed negative correlations with temperature, wind speed, and precipitation, but a positive correlation with relative humidity. Among the examined meteorological variables, temperature showed the strongest association with SO_2_ concentrations (r = −0.47).

Both PM_2.5_ and PM_10_ displayed similar correlation patterns with meteorological factors, showing significant relationships with temperature and relative humidity. Specifically, PM_10_ concentrations were moderate negative correlations with temperature (r = −0.42) and precipitation (r = −0.28). PM_2.5_ exhibited stronger associations, with a pronounced negative correlation with temperature (r = −0.64) and a positive correlation with relative humidity (r = 0.46). These findings indicate that elevated humidity promotes particulate matter accumulation through hygroscopic growth, whereby water vapor condensation increases particle size, while also enhancing secondary aerosol formation from gaseous precursors and reducing dry deposition efficiency. Collectively, these mechanisms contribute to higher ambient particulate matter concentrations. In contrast, precipitation facilitates the wet deposition of fine particles. Furthermore, summer meteorological conditions generally favor particulate dispersion, as higher temperatures intensify atmospheric turbulent mixing and enhancing dilution effects [31].

Unlike the other five pollutants, O_3_ concentrations showed a strong positive correlation with temperature (r = 0.78) and a negative correlation with relative humidity (r = −0.73). This pattern primarily from temperature-enhanced photochemical activity that drives O_3_ production The observed negative correlation with relative humidity stems primarily from its strong inverse relationship with temperature; consequently, high temperatures that favor O_3_ formation coincide with attendant low relative humidity. Existing studies show that when relative humidity drops below 60%, the attenuation of solar radiation by water vapor is weakened, thereby accelerating photochemical reactions [32]. Furthermore, stagnant synoptic conditions, such as uniform-pressure fields and weak baric gradients, inhibit pollutant dispersion and thereby facilitate the accumulation of ozone precursors, notably NOₓ and VOCs. For instance, in southern Xinjiang, meteorological systems like the Central Asian vortex or the periphery of a high-pressure system often induce surface wind speeds below 3 m/s, creating conditions conducive to localized ozone pollution [33].

Overall, low-temperature and high-humidity conditions favor elevated concentrations of CO, NO_2_, PM_10_, PM_2.5_, and SO_2_, aggravating surface-level pollutant accumulation—consistent with established research. During winter months, the low-temperature significantly weakens the atmospheric dispersion capacity, leading to frequent particulate pollution episodes [34]. Although wind speed and precipitation show limited direct influence on pollutant formation, pollution events occur more frequently under low-wind, low-precipitation conditions.

Regarding inter-pollutant relationships, this study systematically analyzed correlations among six criteria air pollutants using their daily monitoring data from 2020 to 2024. Following quality control screening, 1461 complete daily datasets meeting integrity criteria were retained for analysis. The inter-pollutant correlation results are presented in Figure 6. Statistical analysis confirmed significant correlations (*p* < 0.01) among Yining’s primary air pollutants. With the exception of O_3_, all pollutants exhibited positive inter-correlations. The particularly strong correlation between PM_10_ and PM_2_._5_ (r = 0.88) suggests shared pollution sources. The analysis revealed NO_2_ as the predominant precursor influencing particulate concentrations (r = 0.84 with PM_2_._5_), followed by CO (r = 0.78) and SO_2_ (r = 0.57). NO_2_ reacts with water vapor in the atmosphere to form nitric acid (HNO_3_), which subsequently reacts with ammonia (NH_3_) to produce ammonium nitrate (NH_4_NO_3_) [35]. As a key PM_2.5_ component, NH_4_NO_3_ formation is accelerated under high humidity conditions, leading to increased fine particulate concentrations. O_3_ and CO demonstrate a strong inverse relationship (r = −0.62, *p* < 0.01), reflecting the consumption of CO as a precursor in photochemical reactions that generate ozone, thereby explaining their significant negative correlation.

### 3.3. Transport Trajectory Simulation Analysis of Typical Pollution Episodes

**Figure 7:**
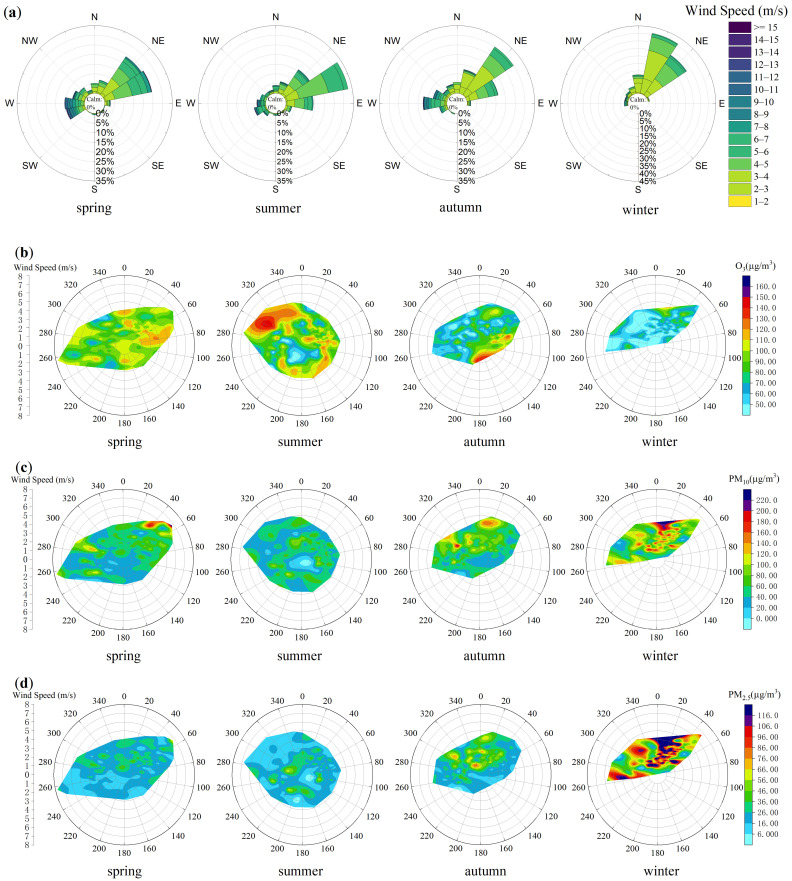
(**a**) Wind rose diagrams for Yining City by season; (**b**) Seasonal polar plots of wind fields versus O_3_ concentrations in Yining City; (**c**) Seasonal polar plots of wind fields versus PM_10_ concentrations in Yining City; (**d**) Seasonal polar plots of wind fields versus PM_2.5_ concentrations in Yining City.

Wind field characteristics critically determine pollutant dispersion, thereby directly influencing transport efficiency and the atmospheric self-cleaning capacity [36]. The study first analyzed wind field characteristics using meteorological data, summarized in the wind rose diagrams (Figure 7a). Subsequent analysis explored the relationships between wind speed and the concentrations of particulate matter and ozone. In the corresponding visualization, Yining City is positioned at the pole, where azimuthal angles denote wind directions, radial distances represent wind speeds, and color gradients reflect pollutant concentration levels, enabling an integrated assessment of wind-driven dispersion effects. Figure 7b specifically depicts seasonal O_3_ variations in relation to wind conditions.

Wind direction analysis reveals a consistent annual prevalence of northeast winds, which maintain stable directions with moderate speeds of 2–5 m/s. During spring, summer, and autumn, southwest winds occur at a relative frequency of 10–20%, often at higher speeds (≥8 m/s) and frequently associated with short-duration, strong wind events, dust uplift, or localized meteorological disturbances. In winter, northeast winds prevail overwhelmingly, with a sharp decline in southwest occurrences and consistently lower wind speeds. This results in a clearly defined seasonal wind regime in Yining, characterized by persistent northeasterly flows punctuated by intermittent yet influential southwest winds.

The results of the relationship between O_3_ concentrations and wind patterns reveals distinct seasonal dependencies. In spring, elevated O_3_ levels are primarily associated with two wind regimes: (1) prevailing westerly winds at 3–4 m/s, and (2) northerly winds at 2–4 m/s. During summer, high O_3_ concentrations show a strong correlation with northwesterly winds at 3–5 m/s. In autumn, O_3_ peaks occur mainly under easterly to southeasterly winds at 2–3 m/s. Cross-seasonal data reveal that the highest O_3_ concentrations consistently occur under low to moderate wind speeds (2–4 m/s) across all seasons. Notably, northwesterly winds contribute substantially to summer O_3_ pollution, likely due to the regional transport zone and its precursors from upwind areas such as Huocheng County.

Figure 7c,d illustrate the seasonal characteristics of PM_10_ and PM_2.5_ concentrations in relation to the wind field parameters in Yining City. The analysis reveals distinct seasonal patterns in particulate matter concentrations. Spring peaks predominantly occur during northeast wind conditions at 3–5 m/s, while autumn shows particulate maxima under two different wind regimes: northwest winds at 2–4 m/s and stronger northeast winds at 4–5 m/s. During winter, the highest particulate concentrations consistently correlate with northerly winds at 3–4 m/s, demonstrating the clear seasonal dependence of air quality on local wind patterns. Overall analysis indicates that elevated particulate matter concentrations in Yining City predominantly occur under northeasterly wind conditions, suggesting that local emissions and regional transport from the northeast direction serve as the primary pollution sources. Notably, upwind areas such as Yining County contribute substantially to particulate pollution in the region.

Based on the identified pollution patterns, this study further investigated a severe PM_2.5_ pollution episode in Yining City during the period 9–12 January 2020, the period with the highest recorded concentrations that month, using HYSPLIT backward trajectory analysis and WRF-model. To evaluate the reliability of the simulation results, WRF simulated meteorological data were compared with observational data from ground monitoring stations during the same period. The model performance was validated using the Mean Bias (MB) and Root Mean Square Error (RMSE) metrics (Table 4). The results demonstrate good agreement between simulated and observed values, indicating that the model effectively reproduced the variations in surface meteorological elements during the pollution episode.

**Figure 8:**
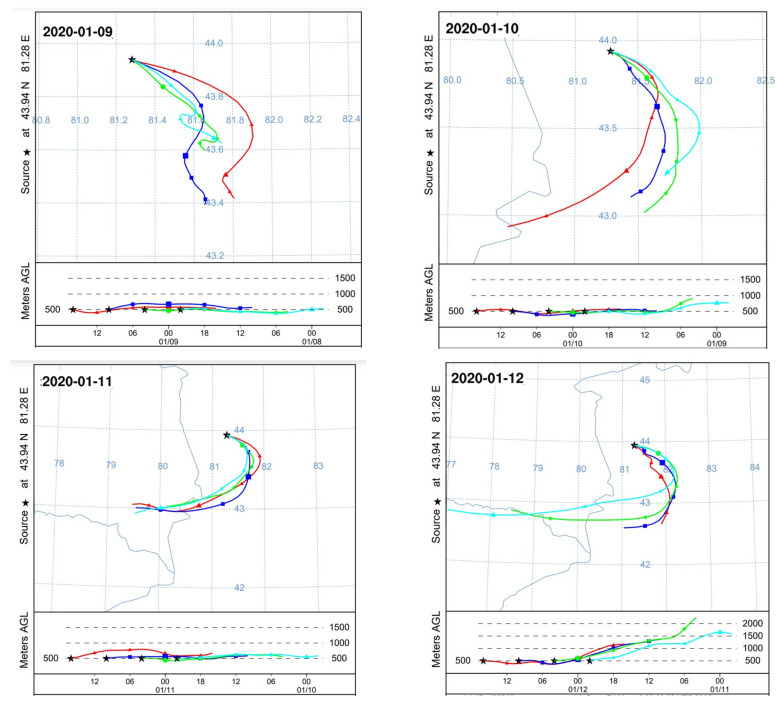
Twenty-four hour backward trajectories of polluted air masses during the period 9–12 January 2020. Colors correspond to different time points: red (16:00), dark blue (10:00), green (04:00), and light blue (22:00).

**Figure 9:**
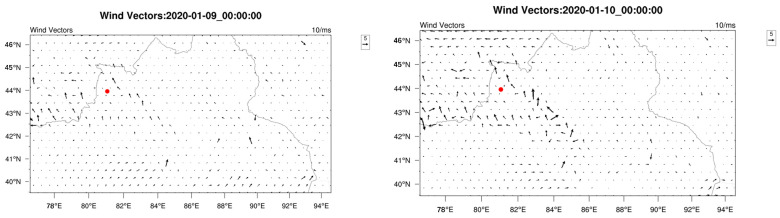

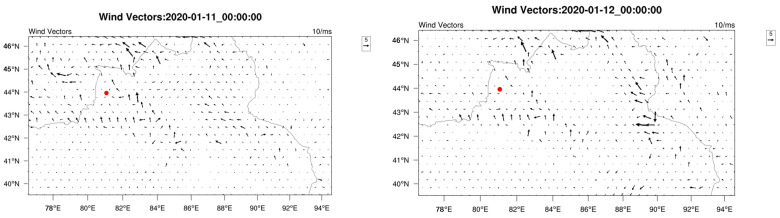
Simulated wind vectors during the period 9–12 January 2020. The red point represents Yining.

To elucidate the potential source regions and transport pathways of a notable PM_2_._5_ pollution event (concentration increase from 164 μg/m^3^ to 256 μg/m^3^), a WRF-HYSPLIT analysis was performed. Figure 8 illustrates the backward trajectory of the polluted air mass, while Figure 9 shows the simulated meteorological conditions, including wind direction and speed, during the pollution episode.

Trajectory analysis reveals that on 9 January, the polluted air mass originating from the southeast significantly influenced on Yining City. This is corroborated by WRF simulation results, which show prevailing southeasterly winds on that day. From 10 to 12 January, air masses consistently originated from the southwest, deflection near 43.5°N latitude, and eventually approached Yining City from southeastern pathways. By integrating topographic data from Figure 2 with WRF-simulated wind fields in Figure 9, distinct orographic wind patterns around Yining City were identified during the study period. The analysis revealed that southwesterly winds prevailed south of the Halqitu–Narat mountain range, while southeasterly winds dominated the northern part of these topographic barriers. The trajectories of polluted air masses aligned closely with these dominant wind directions, indicating significant topographic influence on pollutant transport pathways during the pollution episode.

The results demonstrated that PM_2.5_ concentrations in Yining City were significantly influenced by regional transport under specific meteorological conditions. When calm conditions prevailed in the southern mountains alongside southeasterly flow, enhanced regional transport led to noticeable increases in fine particulate matter levels. More notably, when southwesterly winds dominated the southern part of the mountain range, the transport mechanism incorporated pollutants not only from nearby counties (Yining, Chabuchar, and Gongliu) but also from transboundary sources including Kazakhstan. Under these conditions, particulate pollution intensified considerably, often reaching severe levels.

**Figure 10:**
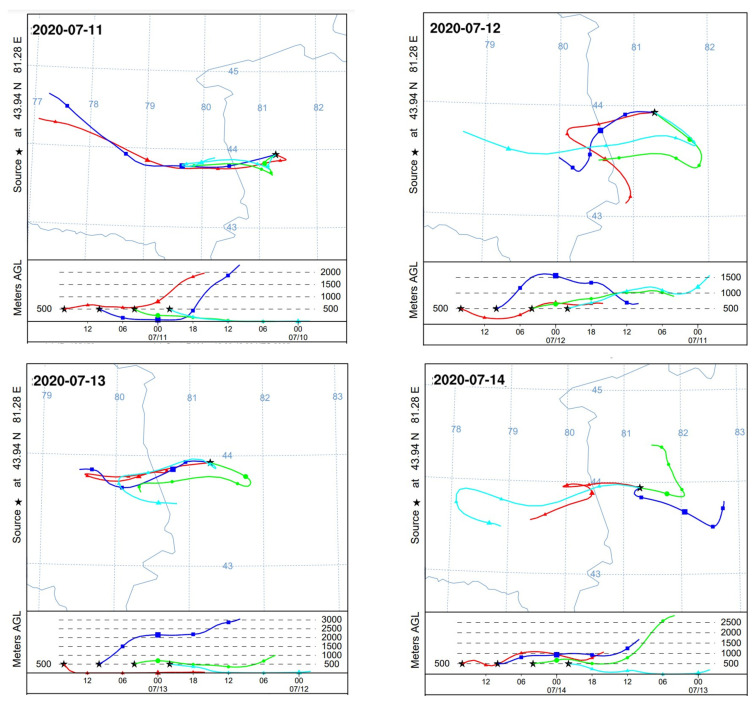
Twenty-four hour backward trajectories of polluted air masses during the period 11–14 July 2020. Colors correspond to different time points: red (16:00), dark blue (10:00), green (04:00), and light blue (22:00).

**Figure 11:**
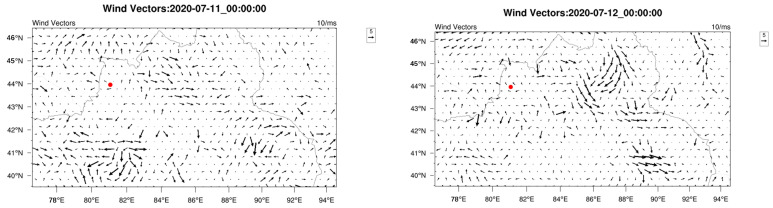

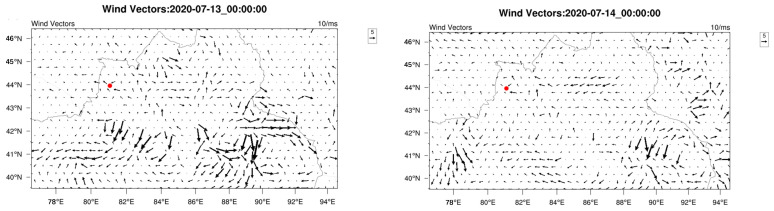
Simulated wind vectors in Yining (11–14 July 2020). The red point represents Yining.

This study also examined a representative ozone pollution episode in Yining City during the period 11–15 July 2020 using numerical simulations. The analysis identified a characteristic variation pattern in O_3_ concentrations, which rose from 116 μg/m^3^ on 11 July to a peak of 150 μg/m^3^ on 14 July before a subsequent decline. Figure 10 presents the backward trajectory analysis of the polluted air mass, and Figure 11 shows the corresponding WRF-simulated wind direction and speed during this episode.

The trajectory analysis revealed distinct transport patterns during the ozone pollution episode. On July 11th, all air masses originated from western directions; however, low wind speeds (<2 m/s) limited their influence on pollutant transport. During the period 12–13 July 2020, while the main trajectories came from the southwest, a secondary transport pathway from the southeast was identified, aligning with the simulated southeast wind patterns on those days. On the peak pollution day (14 July), air masses originated predominantly from the east, coinciding with intensified southeast winds (4–5 m/s) in the meteorological simulations, which enhanced regional transport. The analysis further indicated that air mass transport differentially influenced O_3_ concentrations depending on prevailing wind directions. Southwesterly winds were associated with a moderate transport effect on ozone levels, whereas easterly wind conditions led to a more pronounced influence.

These characteristics suggest that ozone pollution in the study area results from the combined influences of emissions from southwestern and eastern adjacent regions, primarily Huocheng County, Yining County and Gongliu County, with additional contributions from transboundary air masses, including those originating in Kazakhstan.

## 4. Conclusions

This study presents a systematic analysis of atmospheric pollutant monitoring data from Yining City during the period 2020–2024, revealing distinct temporal variations in ambient air quality. By integrating meteorological monitoring data, the study identified the principal factors controlling air pollution in the region. Using coupled WRF and HYSPLIT modeling approach, the study established a pollution source-apportionment simulation system tailored for Yining and its surrounding areas. Numerical simulations were performed to characterize meteorological fields and pollutant transport pathways during representative monthly periods, yielding the following key findings:

1. Yining City showed marked improvement in overall air quality during the 2020–2024 period. The proportion of days meeting national air quality standards for all six criteria pollutants (SO_2_, PM_10_, PM_2.5_, NO_2_, O_3_, and CO) increased from 82.51% in 2020 to 95.6% in 2024. Three-year average concentrations of SO_2_, NO_2_, O_3,_ and CO consistently met the Grade I standards of China’s National Ambient Air Quality Standards (GB 3095-2012) [20], while PM_10_ and PM_2.5_ met Grade II standards. Statistically significant reductions were observed for CO, PM_10_ and PM_2.5_, indicating substantial air quality enhancement. Distinct temporal patterns emerged: NO_2_ and SO_2_ remained relatively stable, whereas O_3_ increased significant in recent years (2022–2024), suggesting enhanced regional photochemical activity. Seasonal variations showed winter peaks for all pollutants except O_3_, which peaked in summer. Monthly profiles revealed a U-shaped trend (January maximum) for SO_2_, PM_2.5_, NO_2,_ and CO; a W-shaped pattern for PM_10_; and a distinct inverted U-shaped profile (July peak) for O_3_;
2. Meteorological conditions critically influence air pollution in Yining. All six pollutants showed significant correlations with temperature and relative humidity. With the exception of O_3_, pollutants mainly accumulated under cold, humid conditions, whereas O_3_ formation was favored by warm, dry weather. Summer northwesterly winds (2–4 m/s) substantially enhance O_3_ pollution, with Huocheng County identified as the primary upwind source region. During winter, northerly winds (3–4 m/s) significantly increased particulate matter levels, largely originating from Yining County;
3. Significant correlations were identified between gaseous precursors (SO_2_, NO_2_, CO) and secondary pollutants (PM_2.5_, PM_10_, O_3_). PM_2.5_ and PM_10_ showed a strong correlation coefficient (r = 0.88), indicating the same sources. Among the gaseous precursors, NO_2_ exhibited the strongest positive correlations with particulate matter (r = 0.84 with PM_2.5_; r = 0.76 with PM_10_). In contrast, CO showed a strongest negative correlation with O_3_ (r = −0.62), suggesting its consumption in photochemical processes. These results highlight NO_2_ as the dominant precursor for particulate formation, while CO plays a key role in ozone depletion;
4. WRF and HYSPLIT modeling results indicated that regional transport significantly affects pollution in Yining. PM_2.5_ pollution events were predominantly influenced by southwestern air masses from Chabuchar County, whereas O_3_ episodes were mainly driven by eastern transport from Yining County and Gongliu County, reflecting distinct spatial patterns for different pollutants.

## Figures and Tables

**Table 1:** WRF model parameter configurations.

Parameter	Configuration/Scheme
Map projection	Lambert
Standard parallels	30° N and 60° N
Boundary conditions	NCEP FNL 1° × 1° reanalysis data (ds083.2)
Microphysics	WSM6 (6-class graupel scheme)
Longwave radiation	RRTM
Shortwave radiation	Goddard
Surface layer	Monin–Obukhov
Land surface	Thermal diffusion
Planetary boundary layer	Yonsei University (YSU)
Cumulus parameterization	Kain–Fritsch (new Eta) scheme for shallow convection

**Table 2:** Annual mean concentrations of major pollutants in Yining City (2020–2024).

Year	O_3_ (μg/m^3^)	PM_10_ (μg/m^3^)	PM_2.5_ (μg/m^3^)	NO_2_ (μg/m^3^)	SO_2_ (μg/m^3^)	CO (mg/m^3^)
2020	82	72	44	30	14	1.4
2021	85	66	36	30	12	1.3
2022	91	62	37	27	10	1.1
2023	91	67	39	31	9	1.1
2024	89	51	28	28	8	0.9

**Table 3:** Percentage of days exceeding the air quality standards for each pollutant.

Year	CO	NO_2_	O_3__8 h	PM_10_	PM_2.5_	SO_2_
2020	6.552%	2.184%	0.000%	9.009%	18.018%	0.000%
2021	3.014%	2.466%	0.000%	4.110%	9.590%	0.000%
2022	3.562%	1.370%	0.822%	5.206%	14.522%	0.000%
2023	4.384%	3.562%	0.274%	6.576%	13.152%	0.000%
2024	0.000%	0.000%	0.273%	0.819%	4.914%	0.000%
Average	3.502%	1.916%	0.274%	5.144%	12.039%	0.000%

O_3__8 h means daily maximum 8 h average O_3_ concentration.

**Table 4:** Evaluation of the WRF model performance for meteorological factors.

Meteorological Factor	Inspection Indicators	2020-01	2020-07
T2	MB	2.78 K	3.05 K
	RMSE	4.14 K	3.87 K
WS10	MB	3.59 m/s	2.30 m/s
	RMSE	4.24 m/s	3.20 m/s
RH2	MB	0.35%	−9.44%
	RMSE	13.18%	15.07%

Meteorological factors: T2-temperature at 2 m, WS10-wind speed at 10 m, RH2-relative humidity at 2 m.

## Data Availability

The datasets used and/or analyzed during the current study are available from the corresponding author upon reasonable request.

## References

[1] Kim K.H., Kabir E., Kabir S. (2015). A review on the human health impact of airborne particulate matter. Environ. Int..

[2] Li T.N., Qiu J.X., Fang C.S. (2020). A brief analysis of the hazards of ozone in the environment and relevant prevention and treatment. World Environ..

[3] Li H.S., Hou X.L., Xue J., Guo T.F., Zou T.S., Zhang H.F., Guo X., Li M.X., Hao J.M. (2023). Practices and Empirical Insights from the National Research Program for Key Issues in Air Pollution in Beijing-Tianjin-Hebei and Surrounding Areas. Engineering.

[4] Yu W.X., Wang Y., Wang H.L., Zhu S.Q., Wang P., Zhang H.L. (2024). Response of ozone to meteorology and atmospheric oxidation capacity in the Yangtze river Delta from 2017 to 2020. Atmos. Environ..

[5] Mai B., Diao Y., Yang H., Deng T., Zou Y., Wang Y., Lan W., Liu X., Deng X. (2024). Assessing atmospheric CO_2_ concentrations and contributions from biogenic and anthropogenic sources in the Pearl River Delta region. Urban Clim..

[6] Han X., Liu Y.Q., Gao H., Ma J.M., Mao X.X., Wang Y.T., Ma X.D. (2017). Forecasting PM2.5 induced male lung cancer morbidity in China using satellite retrieved PM2.5 and spatial analysis. Sci. Total Environ..

[7] Shrestha A., Luo W. (2017). An assessment of groundwater contamination in Central Valley aquifer, California using geodetector method. Ann. GIS.

[8] Min Y.F., Huang W.L., Ma M.J., Zhang Y.N. (2021). Simulations in the Topography Effects of Tianshan Mountains on an Extreme Precipitation Event in the Ili River Valley, China. Atmosphere.

[9] Zhou C.H. (2006). Analyzing atomospheric environment pollution character and improving measure of Yining City. Environ. Prot. Xinjiang.

[10] Li Y., Song X.M. (2008). The temporal and spatial distribution characteristics of heavy fog in Yining. Desert Oasis Meteorol..

[11] Zhou C.H., Liu S.H., Jiapaer Y., Wang S.Y., Guo X.Y., Ding F., Luo J. (2023). Analysis of Air Pollution Characteristics and Countermeasures in Yining City During the “13th Five-Year Plan” Period. Arid Environ. Monit..

[12] Xie Y.X., Tang X., Guo Y.H., Lin C.Y., Wu H.J., Lu M.M., Wang Z.F. (2019). Spatial and Temporal Distribution of Atmospheric Particulate Matter in Xinjiang. Environ. Monit. China.

[13] Yining City People's Government Yining City Ambient Air Quality Deadline Compliance Plan (2021–2025). https://www.yining.gov.cn/yining/c116600/202206/f0410c9a361d418aa7c0f8fe7e9314d7.shtml.

[14] Zhang S.X., Zhang Z.Z., Li Y., Du X.H., Qu L.L., Tang W., Xu J., Meng F. (2023). Formation processes and source contributions of ground–level ozone in urban and suburban Beijing using the WRF–CMAQ modelling system. J. Environ. Sci..

[15] Ge S., Wang S., Xu Q., Ho T. (2021). CAMx simulations of the control of anthropogenic emissions on the reduction of ozone formation in Southeast Texas of USA. Atmos. Pollut. Res..

[16] Lang J.L., Liang X.Y., Li S.Y., Zhou Y., Chen D.S., Zhang Y.Y., Xu L.T. (2021). Understanding the impact of vehicular emissions on air pollution from the perspective of regional transport: A case study of the Beijing-Tianjin-Hebei region in China. Sci. Total Environ..

[17] Wen W., Shen L.Y., Sheng L., Ma X., Wang J.K., Guan C.G., Deng G., Li H.Q., Zhou B. (2025). Impact of meteorological uncertainties on PM2.5 forecast: An ensemble air quality forecast study during 2022 Beijing Winter Olympics. Atmos. Environ..

[18] Yang X.L., Wang Y., Li B., Zhang J.X., Lai X.L. (2018). Influence of Meteorological Elements on Pollutant Concentration in River Valley Terrain. Res. Environ. Sci..

[19] Zhang L.L., Sun H.L., Yang Y.H., Lu B.B., Liu T.Y., Lan X.L., Cao L.J. (2022). Transport characteristics and pollution sources of PM2.5 in Yining City in winter. J. Atmos. Environ. Opt..

[20] (2012). Ambient Air Quality Standards.

[21] Stein A.F., Draxler R.R., Rolph G.D., Stunder B.J.B., Cohen M.D., Ngan F. (2015). NOAA’s HYSPLIT Atmospheric Transport and Dispersion Modeling System. Bull. Am. Meteorol. Soc..

[22] Zhou Y.C., Zhao Y.Z., Gao X., Lei J.Q., Dong D.W. (2025). Dust weather in Tarim Basin derived from a dust event on April 18–21, 2023. Atmos. Pollut. Res..

[23] Haseeb M., Tahir Z., Mahmood S.A., Batool S., Tariq A., Lu L., Soufan W. (2024). Spatio-temporal assessment of aerosol and cloud properties using MODIS satellite data and a HYSPLIT model: Implications for climate and agricultural systems. Atmos. Environ. X.

[24] Wang Y., Stein A.F., Draxler R.R., de la Rosa J.D., Zhang X. (2011). Global sand and dust storms in 2008: Observation and HYSPLIT model verification. Atmos. Environ..

[25] Wei X.J., Zhao X.M., Wang Q., Xiao M.M. (2022). The Analysis of the Influence of Meteorological Factors on PM2.5 Concentration Based on Characteristic Index. Environ. Monit. China.

[26] (2012). Technical regulation on Ambient Air Quality Index (on Trial).

[27] Yu P. (2023). The Memory of Air Pollutants in ChinaRegion. Master Thesis.

[28] Yuval, Tritscher T., Raz R., Levi Y., Levy I., Broday D.M. (2020). Emissions vs. turbulence and atmospheric stability: A study of their relative importance in determining air pollutant concentrations. Sci. Total Environ..

[29] Wang H., Li J.H., Peng Y., Zhang M., Che H.Z., Zhang X.Y. (2019). The impacts of the meteorology features on PM2.5 levels during a severe haze episode in central-east China. Atmos. Environ..

[30] Yi K., Liu J.F., Wang X.J., Ma J.M., Hu J.Y., Wan Y., Xu J.Y., Yang H.Z., Liu H.Z., Xiang S.L. (2019). A combined Arctic-tropical climate pattern controlling the inter-annual climate variability of wintertime PM2.5 over the North China Plain. Environ. Pollut..

[31] Chen Y.L., Wang C., Zhou Y.Y., Liu S.Q., Xiao T.G., Wen X.H. (2020). Study on the Characteristics of Air Humidity and Its Effects on Air Quality in Chengdu. Front. Environ. Prot..

[32] Chen C., Zhang M.X., Liu J., Fang J.L., Guo Y.F., Li T.T., Shi X.M. (2022). Acute impact of ambient fine particulate matter and ozone on daily outpatient visits and its seasonal differences in Beijing-Tianjin-Hebei and surrounding areas. Acta Meteorol. Sin..

[33] Zhang J.L., Li R.Q., Li N., Li H.H., Shi J.J. (2023). Preliminary Analysis of the Water Vapor Characteristics of the “July 19” Heavy Rain in 2021 in the Tarim Basin, Xinjiang. Chin. J. Atmos. Sci..

[34] Gao S.N. (2018). Analysis of the Correlation Between Air Quality and Meteorological Elements in Xi’an City. J. Xi’an Univ. (Nat. Sci. Ed.).

[35] Zuo M., Li G.H., Wu J.R. (2022). Synergetic effects of NO_2_ and SO_2_ on air particulate matter pollution in the Guanzhong Basin (GZB), China. J. Earth Environ..

[36] Cui J.M., Wang T.J., Gao L.B., Cao Y.Q., Wang Q.G. (2020). Observational analysis on air pollution characteristics in Nanjing during winter of 2016. J. Meteorol. Sci..

